# Supplementary material for: Time-series transcriptome analysis identified differentially expressed genes in broiler chicken infected with mixed Eimeria species
Source: Front Genet. 2022 Aug 8;13:886781. doi: 10.3389/fgene.2022.886781 (PMC9393255; doi:10.3389/fgene.2022.886781)
Supplement: Supplementary file 2 [file DataSheet1.ZIP › 7dpi_KEGG.Gsea.1625115961950/KEGG_PHENYLALANINE_METABOLISM.html]

Details for gene set KEGG\_PHENYLALANINE\_METABOLISM[GSEA]

|  || Dataset | TMM\_7dpi\_gct\_format\_7dpi\_gct\_format.Class\_4dpi.cls #PC\_versus\_NC.Class\_4dpi.cls #PC\_versus\_NC\_repos |
| Phenotype | Class\_4dpi.cls#PC\_versus\_NC\_repos |
| Upregulated in class | 1 |
| GeneSet | KEGG\_PHENYLALANINE\_METABOLISM |
| Enrichment Score (ES) | 0.4311277 |
| Normalized Enrichment Score (NES) | 1.2497853 |
| Nominal p-value | 0.17792422 |
| FDR q-value | 0.39336228 |
| FWER p-Value | 0.9834 |
Table: GSEA Results Summary

  

Fig 1: Enrichment plot: KEGG\_PHENYLALANINE\_METABOLISM      
 Profile of the Running ES Score & Positions of GeneSet Members on the Rank Ordered List

  

| SYMBOL | TITLE | RANK IN GENE LIST | RANK METRIC SCORE | RUNNING ES | CORE ENRICHMENT || 1 | PRDX6 | na | 111 | 1.222 | 0.1472 | Yes |
| 2 | GOT2 | na | 227 | 1.009 | 0.2668 | Yes |
| 3 | MIF | na | 317 | 0.924 | 0.3777 | Yes |
| 4 | GOT1 | na | 673 | 0.649 | 0.4311 | Yes |
| 5 | ALDH3B2 | na | 4510 | -0.051 | 0.1180 | No |
| 6 | IL4I1 | na | 4842 | -0.086 | 0.1014 | No |
| 7 | AOC3 | na | 6496 | -0.253 | -0.0040 | No |
| 8 | MAOA | na | 6874 | -0.294 | 0.0022 | No |
| 9 | DDC | na | 9496 | -0.631 | -0.1355 | No |
| 10 | MAOB | na | 11115 | -0.968 | -0.1463 | No |
| 11 | ALDH1A3 | na | 11961 | -1.725 | 0.0041 | No |
Table: GSEA details [plain text format]

  

Fig 2: KEGG\_PHENYLALANINE\_METABOLISM      
 Blue-Pink O' Gram in the Space of the Analyzed GeneSet

  

Fig 3: KEGG\_PHENYLALANINE\_METABOLISM: Random ES distribution      
 Gene set null distribution of ES for **KEGG\_PHENYLALANINE\_METABOLISM**
